# Bridging Lived and Expert Experience: A Qualitative Study Exploring Lived‐Experience Experts' and Researchers' Experiences of Public Engagement in Cancer Research

**DOI:** 10.1111/hex.70848

**Published:** 2026-08-31

**Authors:** Piotr Teodorowski, Beth J. Maclin, Joi Miner, Jonathan B. Green, Daniel G. Garza, Marcus Arana, Edward Duncan, Jonine Figueroa, Sarah S. Jackson, Liz Forbat

**Affiliations:** ^1^ University of Stirling Stirling UK; ^2^ Division of Cancer Epidemiology and Genetics National Cancer Institute Bethesda MD USA; ^3^ Community Advisory Board Stirling UK; ^4^ Marie Curie Edinburgh UK

## Abstract

**Background:**

Public engagement has the potential to make research more impactful, and it has increased in recent years in cancer research. Yet, there is limited understanding of how this process of bringing lived experience into research works, and of what leads to tokenistic and non‐tokenistic engagement.

**Aims and Objectives:**

To explore public engagement in cancer research in the United States from the perspective of both lived‐experience experts and researchers.

**Methods:**

A qualitative study consisting of interviews with 15 lived‐experience experts engaged in cancer research and 15 cancer researchers. Data were analysed using reflexive thematic analysis.

**Results:**

Findings are captured in four themes shared by both participant groups and mapped onto Jürgen Habermas's theory of communicative action, which helped explain the engagement process. First, public engagement was invaluable to research, but also personally and professionally. Second, underlying foundations and principles underpinned the non‐tokenistic engagement process. Third, engagement led to the creation of a partnership between cancer researchers and lived‐experience experts. Fourth, engagement was about creating and enriching culture within a research institution and in the community.

**Conclusions:**

The study provided a better understanding of public engagement in cancer research. Habermas's communicative action theory offered an analytical framework to elicit the importance of mutual understanding in developing common ground between lived‐experience experts and researchers. It highlighted that tensions between lived‐experience experts and researchers, choosing not to act on feedback, and limited institutional support can lead to tokenistic engagement.

**Patient or Public Contribution:**

Four Community Advisory Board members contributed to this study from design through dissemination. They co‐developed topic guides, co‐analysed data, co‐wrote this paper and are named as co‐authors.

## 1 Introduction

Public engagement is internationally recognised as an element of rigorous research [1, 2, 3, 4] and can include shaping research priorities, study design, recruitment, analysis and dissemination [5, 6, 7]. It has the potential to make research more impactful by shifting the focus toward public priorities, improving research quality, assisting with recruitment, and enhancing the interpretation of findings from a public perspective [8]. Engagement can also help with dissemination by making it easier for the general public to understand [5]. Further, there is a moral argument that public engagement can make the research more democratic [9] and has the potential to increase public trust in health policy [10]. However, there are different definitions, wording and blurred boundaries of what engagement is [11, 12]. This paper understands it as an “*active partnership between researchers and the public throughout the entire research process*”, which can take the form of consultation, collaboration, or shared leadership [13]. The term “public” collectively refers to patients, members of the public, their families, and significant others. To recognise their expertise, they will be referred to throughout this paper as lived‐experience experts.

Many fields have experienced increased public engagement, including cancer research [6, 14, 15]. Public engagement in cancer research shares many similarities with other medical fields, yet it has potential to provide new insights [6] even while facing some challenges. First, public engagement in cancer research takes many forms, ranging from consultation to more collaborative approaches, but the majority remains at a consultative level [15, 16]. These consultations usually happen only at an early stage of the research [6]. Thus, they risk becoming one‐off engagements, leaving lived‐experience experts unable to see how their contributions influenced research. Second, there is limited understanding of the public engagement process, as prior literature rarely reports on how to conduct it, identify challenges and provide guidance in cancer research [6, 17].

This project aims to enhance understanding of how to meaningfully engage lived‐experience experts in cancer research. The focus is on the United States (US), as prior reviews highlighted that a substantial proportion of studies on this have been published there; one identified it as a primary contributor [14], and two others as second [6, 15]. Thus, the objectives are:

1. Explore the experiences of researchers and lived‐experience experts around public engagement in cancer research in the US.
2. Understand enablers of and barriers to engaging lived‐experience experts in cancer research.
3. Capture strategies to address engagement barriers.

## 2 Methods

### 2.1 Study Design

The qualitative design aimed for an in‐depth exploration of participants' experiences of public engagement in cancer research. This consisted of semi‐structured interviews with cancer researchers (*n* = 15) who engaged the public in their work and lived‐experience experts (*n* = 15) engaged in cancer research. Capturing the experiences of both groups was crucial to understanding the nuanced picture of public engagement.

### 2.2 Community Advisory Board

The Community Advisory Board (CAB), consisting of four lived‐experience experts, served as co‐researchers on the study from design through analysis to dissemination. Table 1 presents Guidance for Reporting Involvement of Patients and the Public 2 (GRIPP2) [18]. Parenteral with enteral nutrition in the critically ill. *Intensive Care Med*. Jul 2000;26(7):893‐900 CAB members' contributions are discussed throughout the methods section, and the protocol outlining their contributions is published elsewhere [19].

**Table 1:** Completed Guidance for Reporting Involvement of Patients and the Public 2 (GRIPP2).

Section and topic	Item	Notes
1: Aim	Report the aim of public engagement in the study	CAB members helped ensure that the public's views are a core part of this study.
2: Methods	Provide a clear description of the methods used for public engagement in the study	The Community Advisory Board of four lived‐experience experts acted as co‐researchers from design through analysis and dissemination.
3: Study results	Outcomes—Report the results of public engagement in the study, including both positive and negative outcomes	Their involvement shaped: 1. data collection tools‐ designing topic guides, providing feedback on initial interviews to update guides with new follow‐up questions 2. analysis‐ initial coding of one interview from each participant group and defining themes 3. dissemination‐ co‐writing the paper through choosing quotes, summaries of themes and commenting on the draft paper.
4: Discussion and conclusions	Outcomes—Comment on the extent to which public engagement influenced the study overall. Describe positive and negative effects	CAB members shaped the focus from a researcher‐only perspective to the public, ensuring that findings are of importance for both groups working on public engagement.
5: Reflections/critical perspective	Comment critically on the study, reflecting on the things that went well and those that did not, so others can learn from this experience	As this project included CAB members as co‐researchers in qualitative research (for most, it was the involvement in qualitative analysis), this posed workload challenges. For example, the coding process was time‐consuming. To overcome these challenges, CAB members received further reading, used voice‐supporting technologies, and the researcher shared notes after each meeting and provided guidance on what to expect next; thus helping to set expectations. When a new stage of qualitative research was underway, this was preceded by training and examples from previous research projects. The researcher's open‐door policy, being available at any time for further inquiries, helped CAB members ask clarifying questions.

### 2.3 Sampling Rationale and Strategy

The sample size was not fixed; it depended on what the project aimed to capture and could be reflected during the data collection [20, 21]. Public engagement in cancer research in the US varies widely by geography, cancer type, and the community. During data collection, the research team (including CAB members) regularly discussed data collection and quality. Following the concept of “information power” [22] guiding the sample size, 15 interviewees per participant group provided sufficient data to address the research aim and objectives based on data quality.

Researchers were recruited using snowball sampling as those conducting public engagement in the cancer research are not easily identifiable. The recruitment of lived‐experience experts was purposive, targeting those already engaged in this role. However, those undertaking active cancer treatment were excluded because the participation would be an unnecessary burden.

### 2.4 Recruitment Procedures

The research team developed the invitee list of potential researcher participants based on existing contacts and a review of the published literature and institutional websites. As the data collection progressed, researcher participants recommended other researchers.

For lived‐experience participants, existing public and community advisory groups in the US were asked to share an invitation with their members. The list was based on National Cancer Institute‐designated cancer centres, as these have established public engagement [23] and recommendations from researchers. Both participant groups received an information sheet detailing the study, and it was the individual's decision to contact the research team to sign up as a participant.

### 2.5 Study Participants

To maintain participants' confidentiality, only group‐level demographic data is reported [24]. Researchers represented a range of disciplines, cancer expertise and spread around the country. Their experience of public engagement in cancer research spans from involving lived‐experience experts in their ongoing doctoral research through decades of research experiences. Disciplines included: population science (*n* = 8), epidemiology (*n* = 3), clinical (*n* = 3), health services research (*n* = 1), public engagement lead (*n* = 1) and other (*n* = 1). Almost half of the participants worked in any type of cancer (*n* = 7); other areas included breast (*n* = 5), cervical (*n* = 2), gynaecological (*n* = 1), testicular (*n* = 1), prostate (*n* = 1), paediatric (*n* = 1), and colorectal (*n* = 1) and other (*n* = 1). The primary locations of research institutions were Southeast (*n* = 4), Midwest (*n* = 4), Northeast (*n* = 3), West (*n* = 3), and Southwest (*n* = 1).

Most lived‐experience experts were engaged in more than one cancer project, had a history of cancer (as survivors or family members), came from various racial and ethnic communities (e.g., Latino, African American, Native American, and White), and contributed to research projects from the East Coast to the West Coast and Hawaii. Their engagement roles spanned a broad spectrum (e.g., community advisory boards, co‐investigator, and co‐lead) with broad years of experience, from recently becoming a lived‐experience expert to long‐term contribution through different research projects over years.

### 2.6 Data Collection

Following the framework for the development of a qualitative semi‐structured interview guide [8] two topic guides were developed, one for each participant group. These are available in Appendices 1 and 2. Questions were based on previous literature and consultations with CAB members. The draft topic guides were field‐tested with participants and then brought back for discussion with CAB members, who identified additional follow‐up questions.

PT conducted interviews on MS Teams or by phone, which were recorded, transcribed and anonymised. PT took field notes during data collection, with his critical reflection on any new data [25], and the anonymised summary was shared as part of updates during CAB meetings. Interviews were between 30 and 60 min. Lived‐experience experts received a $50 shopping voucher as a thank‐you for their time. Researchers did not receive any financial compensation.

### 2.7 Analytic Framework

Research on public engagement remains theoretically underdeveloped [26]. Thus, this paper draws on the work of Jürgen Habermas and his theory of communicative action [27]. His theory has two components: communicative action and strategic action. Communicative action is the idea that interaction among two or more parties can lead to mutual understanding and consensual agreement, which can result in agreed‐upon action. In the context of public engagement in cancer research, these two parties are the public and cancer researchers. Through public engagement, they understand each other better, leading to higher‐quality research, greater focus on public needs, and increased research legitimacy. In contrast to communicative action, strategic action focuses on achieving success rather than understanding others. Within public engagement, it can occur when researchers engage lived‐experience experts because it is a funder requirement (but researchers do not intend to implement their feedback), use academic or medical jargon (thus limiting two‐way communication), or conduct engagement activity in a way that confirms the researcher's initial thoughts.

There are two different relationships between parties. First is what Habermas describes as the lifeworld. In terms of public engagement, this concerns lived experiences, cultural meanings, and individual perspectives. The second is the system in which action is guided through institutional steering (linked to the economy and state power). Examples of these in public engagement include research practices, medical knowledge, institutional policies and funding. Both the lifeworld and the system should co‐exist as they serve different purposes. However, there is a risk of the system “colonising” the lifeworld through strategic actions such as ignoring lived experience or engaging in tokenistic practices that ask for engagement but researchers or institutions do not take it seriously or fail to act upon it [28]. If colonisation occurs, it could lead to poorer‐quality research, a narrower focus on public needs, and reduced public support.

The theory of communicative action has previously been used to explore public and expert expertise in public health [29], public engagement in the development of a health charter [30], cancer research network [31] and to design an evaluation framework for public engagement to assess whether engagement activity was more focused on the organisation or on public concerns [32, 33]. However, Habermas's theory of public engagement is not without criticism. Hodge [34] argues that communicative action is unrealistic to be achieved in its pure, idealistic form in practice, yet it can help promote new ways of working to improve public engagement. Despite that criticism, applying the theory of communicative action in this study is appropriate, as it supports a better understanding of the quality of public engagement in cancer research, communication between lived‐experience experts and researchers, and the relationships between them.

### 2.8 Data Analysis

Data were analysed using reflexive thematic analysis [35, 36] with input from the CAB at each stage of the analysis to improve rigour [37, 38], offering CAB members a strong voice in the discussion [39]. Training was provided to CAB members to ground them in qualitative analysis [40]. PT provided an overview of the analysis process, outlining each step and conducted a coding exercise on a sample interview from another project.

The coding was inductive. PT and CAB members coded one interview from each participant group to develop initial codes. Afterwards, PT uploaded all interviews to NVivo 20 to facilitate the analysis. He took the lead in coding all interviews with critical friend [41] input from BJM, who reviewed all coding and met regularly to discuss the coding process. The coding tree was brought back to the CAB meeting, which led to the organisation of codes into candidate themes. These were further refined during subsequent meetings, with all authors agreeing on the final version. The final themes were mapped under two components of Habermas' theory: communicative and strategic actions to bring the analytic framework into the analysis.

### 2.9 Reflexivity

Positionality influences how one interprets data within existing assumptions and biases [36, 42]. PT is an experienced public engagement researcher who works especially with lived‐experience experts in complex research areas (such as data research). LF has interests in public engagement and cancer care. ED's work focuses on complex health interventions, and he has interests in public engagement. PT, LF and ED are based in the UK. SSJ, JF and BJM are cancer researchers with public engagement experience and are based in the US. CAB members represented the public perspective in the data as lay members of the public with prior experience serving as lived‐experience experts on cancer research projects in the US.

### 2.10 Ethics

This project received ethical approvals from the General University Ethics Panel at the University of Stirling in the United Kingdom (reference number GUEP 2025 23682 18392) and the National Cancer Institute Ethics Review Panel in the United States (ref number 25G009‐02). All participants received a consent form before the interview. They completed it on their own time, were encouraged to ask questions and had to send it back to the researcher.

## 3 Results

All participants shared rich experiences of public engagement in cancer research. These took place in various contexts and locations and came from both researcher participants (RP) and lived‐experience participants (LEP). Considering the perspective of both participant groups enabled a more comprehensive understanding of public engagement; thus, these are presented in four joint themes. Themes: (1) engagement is invaluable, (2) foundations for introducing lived experience into research, (3) constructing partnerships reflect a lifeworld perspective, mutual understanding and joint working; thus, they were mapped under communicative actions. The fourth theme of enriching culture reflects on system pressures and the risk of colonisation of the lifeworld; thus, it is mapped under strategic action.

### 3.1 Communicative Actions

#### 3.1.1 Engagement Is Invaluable

Public engagement was invaluable to the participants, not only for improving the research but also for offering unexpected professional and personal pathways. For researchers, it provided a fresh perspective on their work, offered an opportunity to learn something new about public perspectives, and reassured them of the importance of their research:

I honestly was surprised about how enthusiastic the [Community Advisory Board] members were about my research question. That was really just affirming, and it felt good to me as a researcher to have that excitement. I wasn't expecting that to be such a big benefit to me, but it made me a lot more confident in the work that I was doing.

(RP15)

For some researchers, it was inspirational and became a motivation to continue in their field:

It's been refreshing. It makes you continue to love the work that you do.

(RP3)

Lived‐experience experts saw their contribution as making a difference by leading researchers back on track. This difference did not have to be substantial; even if it meant making only a small impact on the overall project, they found it important. Researchers recognised that the challenge to their worldview by lived‐experience experts was invaluable and shaped the project's quality, reduced research waste, saved time, shifted focus into public needs, created new connections with the broader community (leading to successful grant applications) and, in some cases, was the only way to make projects successful:

[Lived‐experience experts] downright hated [the research project], but all of their feedback was right on point, so there's no way we could have taken this [research] forward. It would have gone over like a lead balloon, like nobody's going to do it, so this feedback that we get from our community members is absolutely critical to success.

(RP11)

For lived‐experience experts, public engagement became invaluable in shaping them as individuals through sharing, reflecting, and learning. For one Indigenous lived‐experience expert, engagement allowed them to reconnect with their cultural background and history, as they felt they needed to better understand their own community before raising their voice with the research team.

For most lived‐experience experts, public engagement offered an opportunity to learn new skills, put themselves out there (e.g. through co‐presenting research and co‐authoring papers) and become “*A PhD in me, my tumour, and the science of cancer*” (LEP10), even for those who felt that they were not good with science at school. Education about research and understanding new medical/research terms often surprised participants with their learning skills. This became rewarding on an individual level and, for some, engagement shaped their professional career as they became inspired to sign up for a postgraduate degree, mentioned a public engagement role on their CV, and even linked new skills to new job opportunities:

I added it to my resume. (…) I think it helped me get a role with the [organisation], [organisation 2], and now I'm with [organisation 3] (…) I think it just completely changed the trajectory of my career. (…) It gave me the confidence.

(LEP15)

Researchers learn from the lifeworld (represented by lived‐experience experts); in turn, lived‐experience experts learn from researchers, and this communication improves the research project. However, the process of integrating lived experience into research is not self‐generating; it requires a strong foundation, which is explored in the next theme.

#### 3.1.2 Foundations for Introducing Lived Experience into Research

Inviting individuals with direct knowledge and experience of a research project's subject matter to engage with the researcher introduces lived experience into the research. Researchers “*can't have a one‐size‐fits‐all”* (RP4) approach for public engagement, but they can have a menu of strategies to use:

We wish we could just have one and it serves for every need, but there's no way it will. The more tailored you have them, so the more distinct and unique they are to the specific topic, the more rich your information from them is going to be.

(RP4)

However, flexibility does not mean that there are no basic foundations and principles underlying the engagement process. These are captured in two subthemes. The first subtheme, “desire to improve the community”, explains why lived‐experience experts and researchers want to work together as partners on research projects. The second, “transforming engagement into a resource”, shows how to make it a meaningful process for both researchers and lived‐experience experts.

##### 3.1.2.1 Desire to Improve the Community

The main reason lived‐experience experts and researchers worked together was their shared desire to improve communities and cancer outcomes. For lived‐experience experts, that was often linked to their experience of cancer diagnosis (or their loved ones), seen as a desire to “*lend [their] voice*” (LEP3) to research. For some, it was about using the contribution to say thank you for the care they received (especially if they felt they received exceptional care or were lucky to access free or novel treatment) or to make the experience better for others:

Well, it was in part to honour someone that I once was in love with, (…) had a battle with cancer (…). So, it's part of me honouring his memory and doing what I said I would do, try to get the word out, raise awareness as best I could. Even though I'm one of several people that are a part of the [community advisory board], it's our collective work that can change and maybe save some lives.

(LEP14)

Public engagement was about doing it for the community and not for money. This desire to improve communities meant that, for many, contributing as lived‐experience experts was more important than being reimbursed for their time:

When we lost a bunch of funding (…) we had to let the [advisory board] know, and we had to say, we're shifting to quarterly. Many members came forward and said, I would donate my time, don't pay me a stipend, I want to continue.

(RP5)

##### 3.1.2.2 Transforming Engagement into a Resource

The participants spoke about training opportunities, the importance of lay language, communication between researchers and lived‐experience experts, and ensuring that the discussion is welcoming to all who are part of it. Taken together, these issues form a foundation for transforming engagement into a meaningful resource for both sides.

Lived‐experience experts were often unfamiliar with the science and biology of cancer, the research process, or the topic under study (beyond their lived‐experience perspective). This meant that in order to be able to contribute in a non‐tokenistic way, researchers offered inductions and training opportunities so lived‐experience experts could understand more about research; this could have “*been both high level and getting to be up close with some of the details and being walked through every single part of the process*” (LEP4). This included the recruitment process, ethics applications, retention, results or topic‐specific information (e.g., genetic biospecimens). It was supposed to provide a foundation for discussion. One example of a learning opportunity embedded within the project was a journal club:

What we call the journal club, where they would put together some articles for us to read and discuss. That was always an extra thing, we would sometimes learn different topics that could be helpful.

(RP15)

Use of lay language and the avoidance of scientific and medical jargon were linked to training opportunities. Participants often perceived cancer research as having complex terminology, which they felt required additional steps to overcome when communicating:

There are a lot of people who just have lived experiences, cancer patients who aren't familiar with the research terminology. So, it's important to make it as accessible as possible and to break things down as much as you can to make sure you are not leaving anyone behind.

(RP15)

Creating a dictionary or glossary of complex terms was recommended to allow lived‐experience experts to refer to it when needed, rather than keep asking researchers to remind them what it means. However, where necessary, lived‐experience experts felt open about asking for clarification:

[S]ome of it did have some technical terms. And so I questioned it and asked for explanations, and [researchers] were very good about breaking it down. And then also made a note that, okay, that part's a little technical, so maybe we ought to break it down a little more for the future.

(LEP8)

The last foundation of transforming engagement into a resource is the creation of an inclusive and welcoming space for discussion, characterised by respect for everyone, where people feel heard rather than hostile. Lived‐experience experts were “*encouraged to go ahead and speak up what we're thinking. There is no right or wrong input*” (LEP8). Respectful space encouraged them to raise their voice and challenge researchers when they disagreed:

I've noticed over time, with building that relationship, that they reach out more and they want to engage more. And they are speaking up more at the meetings. And I think that's also important, you know? They're not afraid to talk to researchers and ask them really hard questions. Or critique.

(RP13)

Only when the foundations are embedded can communicative action take place, with lived‐experience experts and researchers constructing partnerships.

#### 3.1.3 Constructing Partnerships

Constructing partnerships was seen as central to public engagement in research. These relationships were integral in generating invaluable information for cancer studies. Knowing the availability and other needs of the population of focus and keeping active connections with the community, such as inclusion in the research itself, findings, and presence within the communities, was a make‐or‐break requirement for researchers' ability to acquire accurate findings and help the communities of focus feel valued and valuable. Two subthemes elicit the partnership construction process. The first one, “avoiding helicopter research,” focuses on what makes a partnership genuine, and the second, “disconnect,” explores what happens when there is a disconnect between researchers and lived‐experience experts.

##### 3.1.3.1 “Avoiding Helicopter Research”

This subtheme elicits the perspective that engagement is a process maintained throughout a study and requires continued presence, with the needs of both researchers and lived‐experience experts prioritised. Helicopter research is the opposite with regards to community engagement:

Helicopter research is when either an institutional researcher, academia or wherever, they leave their place, they fly out, drop down into a community, collect data and go back and are never seen again.

(RP8)

Both participant groups discussed public engagement activities that occur both online and in person. However, in‐person meetings had a stronger impact on building connections between people as they allowed them to establish deeper rapport:

When you meet someone live in the flesh and you have a little time to get to know them behind the scenes, it's just a gamechanger and …it's going to make me cry, but I have so much respect for everybody I met.

(LEP9)

For researchers, getting closer to the community meant learning about the population they study, including its needs, perspectives, background, and history. This applied to both researchers who studied communities different from their own and those with insider perspectives (if they considered themselves part of the community), as intergroup differences made understanding local needs necessary. For some researchers, it was feeling like an early‐career investigator “*all over again, every single time, because it's a different community, it's a different group of people, and I can bring my wisdom of how to do it, but every community's different*” (RP11). This attitude was linked to being humble about what lived‐experience experts might share. Many lived‐experience experts appreciated that approach and recognised it as needed:

There are historical things that have happened that may cause mistrust or trepidation as far as people joining [community advisory group meetings]. And so we're encouraged to say how that might be perceived from our culture, from our ethnicities.

(LEP8)

If the partnership was already established and lived‐experience experts contributed to the research, both participant groups felt the need to continue learning and adjusting the partnership. They spoke about how lived‐experience experts provide formal and informal feedback through surveys, interviews, and discussions on how to improve the public engagement process (e.g. whether lived‐experience experts understood everything, whether there are different ways to discuss this topic and what else should happen in the research):

Being open to hearing the feedback and making changes that will benefit or hopefully make the group run more smoothly. I think the other advice is make sure you're truly ready and willing to listen and act on the feedback.

(RP13)

Researchers noted that building the connection can be time‐consuming, and one should not expect engagement to yield a specific research outcome at the outset, except to build trust for a future partnership. That issue of trust was seen as a connection between the researcher as an individual and lived‐experience experts, and should not be considered the same as the connection with the research institution:

Be willing to give of yourself before you are expecting to get anything in return (…) people have to see you as a trustworthy partner. Even when they don't think your institution may be the most trustworthy, and so I've seen that happen, where they were, like, ‘I'm not sure about the institution per se, but I think you're a trustworthy person so I'm willing to engage with you'.

(RP2)

##### 3.1.3.2 Disconnect

Although most participants reported that public engagement improved cancer research, many described challenges in the partnership between researchers and lived‐experience experts, including disagreements. This was especially evident in each group having different expectations:

I felt like sometimes people would say things and it was outside of the purview of what we could do. I felt like, which feels very elitist to say, but I felt like, you don't understand the research question or you don't understand what we're trying to do and we can't do that.

(RP12)

Those on the advisory board didn't feel like they were being heard or utilised or valued, and so in our discussions, it was learned that, well, it's not that they weren't but the researchers or the scientists didn't really know what our purpose was and didn't understand how to use us and what to do.

(LEP5)

One participant reflected on how to respond if feedback from lived‐experience experts could not be implemented, recognising that one must handle it well by clearly stating why it cannot be done and the reasoning behind the decision. Lived‐experience experts accepted that sometimes the science would come first, “*based on science and what is correct and what is safe*” (LEP10). They recognised that it was not always possible to implement all their feedback; others recognised that they might not be so attached to their suggestions, but it would be good to know why they were not implemented. Researchers noted that training lived‐experience experts is helpful, yet lived‐experience experts do not (and cannot) control everything, and it is not always possible to meet everyone's priorities (especially when there are disagreements within the community of focus).

The creation of a non‐tokenistic partnership entails communicative actions, but it also presents challenges. What could influence this relationship between researchers and lived‐experience experts is the system within which cancer researchers work; thus, there is a need to explore research culture.

### 3.2 Strategic Actions

#### 3.2.1 Enriching Culture

This theme is about creating an environment where engagement is not a tick‐box exercise but rather a genuinely valued experience for researchers, lived‐experience experts, and the community. The first subtheme, “research institutions”, explores the role of systems in public engagement. The second, “community,” illustrates how lived‐experience experts can feel part of the system.

##### 3.2.1.1 Research Institutions

Participants described public engagement as confidence‐building but sometimes under‐supported, increasing one's workload, and constrained by funding. Researchers valued training and flexible guidance. Despite labour demands, engagement was highly rewarding for all involved due to meaningful relationships, a shared purpose, and improved research quality.

The ideal role of research institutions is to enrich the culture of engagement; however, institutional practices differed widely. Researchers spoke about being offered training, mentoring, and resources on conducting public engagement. The emphasis was on providing a real‐life example of how to do engagement. They felt this was particularly relevant for early‐career researchers:

Particularly for early‐career or anybody, having access to modules, having access to training, having access to, sort of, the evidence base and the science of it all, I think will boost confidence. Just engaging with community members and realising that they're not as scary as you think, I think it's easier for us to talk to other academics, rather than community.

(RP11)

Researchers appreciated when senior leadership and their line managers supported their public engagement work (e.g. through funding). Most recognised that research institutions and funders were currently allocating insufficient funding to reimburse lived‐experience experts for their time. For some researchers, public engagement became a workload challenge that was not necessarily accounted for in their time; for others, it was. For one participant, engagement was on top of other responsibilities:

[Public engagement] was just like, this is something I want to do, I'm going to do it in my free time. Then I was lucky enough that my PI didn't have a problem with it, but it was definitely extra work on top of my normal research workload.

(RP15)

##### 3.2.1.2 Community

Cultural enrichment also happens in the community. Most participants spoke about making lived‐experience experts feel valued, useful and respected when contributing to research:

I had imposter syndrome for quite a while. There are a lot of people in that [Community Advisory Board] that have a lot of community background, advisory board background, but I came in just as my own voice (…) But the researchers in the [Community Advisory Board] have been so incredibly supportive that it's really been one of the best experiences I've had, like, one of the things I'm most proud of.

(LEP1)

The feeling of being a part of the research team was important to lived‐experience experts. One participant spoke highly of the opportunity to address the large project leadership team, and the senior investigator remembered their name and kept referring to what had been shared. Co‐presenting findings at events made lived‐experience experts feel particularly valued. Communication between lived‐experience experts and researchers led to closer relationships and greater trust. Often, moving beyond partnership and trust and becoming individual friendships:

That is around a kinship or friendship or just a connection with someone that's willing to help you think about these problems, and bring their own personal experience.

(RP14)

## 4 Discussion

### 4.1 Overview of Findings

At the empirical level, the findings provide a better understanding of public engagement in cancer research. To the authors' knowledge, this is the first qualitative study to directly explore the perspectives of researchers and lived‐experience experts on public engagement in cancer research. These capture much‐needed information on the impact of engagement on research projects, researchers, and lived‐experience experts, identifying the foundations for implementing public expertise in research, constructing public‐researcher partnerships, and improving the research environment for public engagement. Although new in cancer research, these findings are unsurprising, as many align with other public engagement literature [43, 44, 45]. Key recommendations drawn from the findings are presented in Table 2. This paper's novel contribution to the public engagement literature is theoretical; drawing on Habermas' theory of communicative action, it elicits and highlights the processes that enable lived‐experience experts and researchers to work together to embed public expertise in research.

**Table 2:** Key strategies for non‐tokenistic public engagement.

Researchers and lived‐experience experts actively build shared learning practices and continually aim to learn from each other during the engagement process.
Researchers should develop personal connections with lived‐experience experts; in‐person meetings could facilitate partnership building.
Public engagement should be evaluated on an ongoing basis to improve its quality.
Keep open and honest communication. When lived‐experience feedback cannot be implemented, this should be explained how and why this is the case.
Researchers and lived‐experience experts should share power, control, and influence over research agenda.

### 4.2 Communicative Actions

The study provided numerous examples of communicative action and how it can be achieved. This stands in contrast to Bissell and colleagues' [31] work on cancer research in England, which, also drawing on Habermas's work, found that public engagement focused on system priorities rather than lived expertise. Here, public engagement was about learning from each other, and building shared understanding, with researchers bringing lived expertise (from the lifeworld) to the research world (the system). This could be achieved through bi‐directional partnership schemes in which cancer researchers partner with lived‐experience experts for a couple of months to learn from each other [46]. Both participant groups justified their views with reasons, often showed openness to being persuaded to another viewpoint and responded well to others' arguments (even if they did not necessarily agree with what others wanted to see in the research project). However, previous literature also suggests caution in engaging lived‐experience experts in certain parts of the research process, such as data collection as it might not lead to better quality of research [47].

Communication, previously recognised as an important aspect of public engagement [48], led to the construction of partnerships, thus becoming a cornerstone of the engagement process; this study suggests that it can often be developed on a personal level between individual lived‐experience experts and researchers. Meeting in person can further enhance the construction of a partnership. The participants emphasised the importance of meeting in person to build connections. The COVID pandemic has shifted public engagement activities online, with many lived‐experience experts adapting well to that change [49], but the study by Jones and colleagues [49] showed that the majority of their participants prefer a mix of in‐person and online. This study highlights how in‐person engagement can construct partnerships and that researchers should not ignore it. This may be more challenging in some parts of the globe due to their geography.

The participants recognised the value of collecting feedback from lived‐experience experts on the quality of the engagement process. This aligns with most public engagement frameworks, which recommend ongoing evaluation [50]. However, this study's findings align with a recently identified challenge in a review of public engagement evaluation: researchers usually evaluate engagement, and lived‐experience experts act as co‐evaluators only sometimes [51]. Thus, there is a need for greater power‐sharing with lived‐experience experts as equal co‐evaluators to ensure that both groups jointly decide whether the engagement process works.

### 4.3 Strategic Actions

Communicative actions were the most popular among the participants, yet there were also instances of strategic action in which tokenistic public engagement could occur. The engagement meetings were, in most cases, led by researchers, which did not necessarily mean they would translate into strategic actions, but it increased the risk that researchers would steer the focus toward their priorities.

One example of strategic action is colonisation of knowledge. Previously, researchers in gynaecologic oncology raised concerns about public engagement, fearing it could identify issues irrelevant to clinical research [52]. Some of the researcher participants encountered situations in which they felt public feedback was unhelpful, and they decided that their own medical/research perspective should take precedence over lived‐experience perspectives. This overruling of lived‐experience expertise was an example of system (institutional) colonisation of knowledge, which also raises the question of whether lived‐experience experts were appropriately trained to provide meaningful feedback to researchers. Training in research methods and project‐specific knowledge has been recognised before as an important part of the engagement process [16, 48]. Even after explaining research methodology in lay terms, researchers might feel that lived‐experience experts still do not fully understand the research process [53]. However, the complexity of the topic should not be used as an excuse to avoid public engagement [48]. Tensions between researchers and lived‐experience experts were noted in previous studies [53], and the disconnect subtheme expanded on this issue, capturing the public acceptance that research/medical expertise can overrule lived expertise. This raises the question of when system colonisation can happen and who should decide whether it is appropriate. One way to reduce the harm of colonisation and maintain the relationship is to explain how and why researchers make decisions.

Institutional changes, both bottom‐up and top‐down, are needed for non‐tokenistic public engagement [54]. The review on the impact of public engagement on people involved recognised that researchers face difficulties implementing non‐tokenistic engagement due to a lack of financial resources and time [55]. The findings suggest that the system of institutional norms and support can impact whether and how public engagement looks. Individual researchers can be open to lived expertise, but without institutional buy‐in, the engagement process risks becoming tokenistic. In the US, researchers can follow the Patient‐Centered Outcomes Research Institute's (PCORI) guidance on conducting engagement [56], but more localised guidance and support are needed at the state and institutional levels, for example organisations could provide introductory training in public engagement for cancer researchers [57].

### 4.4 Limitations

The findings are limited only to the US sample. However, key lessons on how lived‐experience experts and researchers can work together would apply to other contexts. Participants come from across the country and focus on different types of cancer, but recruiting only those already involved in public engagement could affect the findings on the role and impact of public engagement in cancer research, thus suggesting the risk of participant bias. It is known that some researchers can be apprehensive towards public engagement [58]. In the US (in contrast to other countries like the United Kingdom), public engagement is not required by all major research funders, suggesting that cancer researchers engage the public in their work largely because they believe in it rather than because they are required to do so to secure funding. Future research should explore the views of researchers who do not want to (or cannot) engage lived‐experience experts in their research, as well as those of lived‐experience experts who have had negative experiences with public engagement.

Excluding lived‐experience experts as potential participants who were undertaking active cancer treatment could have led to perspective bias, as those undertaking treatment might have had different views. However, at the ethical approval stage, it was deemed that excluding them would prioritise participants' wellbeing, as the participation of cancer patients in this study would not have directly benefited the treatment pathway yet could lead to unnecessary fatigue. No interested potential participant was excluded because of this.

Some participants spoke about ongoing public engagement activities; others reflected on past experiences. For some researchers, their public engagement experiences may have spanned over decades. This suggested the potential for recall bias; to mitigate that risk, the topic guides consisted of open‐ended questions to allow space for reflection rather than simply agreeing with the researcher.

## 5 Conclusion

This study explored how to conduct public engagement in research, using cancer research as an example. Using Habermas' communicative action theory, the findings provide deeper insights into the experiences of both the researchers and the lived‐experience experts. Communicative action highlighted the importance of mutual understanding to develop common ground between lived‐experience experts and researchers. Whereas strategic actions emphasised issues such as tensions between lived‐experience experts and researchers, choosing not to act on feedback and how limited institutional support can lead to tokenistic engagement.

## Author Contributions

**Piotr Teodorowski:** conceptualisation, methodology, formal analysis, data curation, investigation, funding acquisition, writing – original draft, writing – review and editing, project administration, resources, software. **Beth J. Maclin:** formal analysis, writing – review and editing. **Joi Miner:** writing – original draft, writing – review and editing, conceptualisation, methodology, formal analysis. **Jonathan B. Green:** conceptualisation, methodology, formal analysis, writing – review and editing, writing – original draft. **Daniel G. Garza:** writing – review and editing, formal analysis, methodology, conceptualisation, writing – original draft. **Marcus Arana:** conceptualisation, methodology, formal analysis, writing – review and editing, writing – original draft. **Edward Duncan:** conceptualisation, supervision, methodology. **Jonine Figueroa:** conceptualisation, supervision, methodology, writing – review and editing. **Sarah S. Jackson:** conceptualisation, supervision, methodology, writing – review and editing. **Liz Forbat:** conceptualisation, supervision, methodology, writing – review and editing.

## Ethics Statement

This project received ethical approvals from the General University Ethics Panel at the University of Stirling in the United Kingdom (reference number GUEP 2025 23682 18392) and the National Cancer Institute Ethics Review Panel in the United States (ref number 25G009‐02).

## Conflicts of Interest

The authors declare no conflicts of interest.

## Supporting information


Supporting File 1

Supporting File 2

## Data Availability

The data that support the findings of this study are available from the corresponding author upon reasonable request.
