# Supplementary material for: Bridging Lived and Expert Experience: A Qualitative Study Exploring Lived‐Experience Experts' and Researchers' Experiences of Public Engagement in Cancer Research
Source: Health Expect. 2026 Aug 31;29(5):e70848. doi: 10.1111/hex.70848 (PMC13527529; doi:10.1111/hex.70848)
Supplement: Supplementary file 1 [file HEX-29-e70848-s001.docx]

**Semi-structured topic guide for researcher participant interview**

*The main questions are numbered with optional prompts in italics.*

**Warm-up discussion**

1. Could you tell me a bit about your cancer research?
2. How did you engage the public or patients in your research?

*How extensive was their engagement?*

**Working with the public**

3. Could you tell me how you set up your community/public advisory board?

*Any internal processes, recruitment, and induction process.*

4. Could you tell me what your patient and public engagement looks like?

*E.g. one-off or multiple meetings, meeting length, stage of study (design, data collection, etc), incentives, setup (e.g. in-person, online, group discussion, survey), who runs engagement activities, and feedback to the public*

5. Have you faced any challenges when engaging members of the public?

*E.g. managing everybody’s schedule, preparation.*

*Would you have done anything differently?*

6. How do members of the public's backgrounds and beliefs influence your engagement strategies? (e.g. financial, education background, race, language skills, personal beliefs)?

*How would these link to your personal background and experiences?*

*How do you ensure everyone’s lived experiences are included and respected?*

*How does people’s background influence the way you run the meetings? (e.g. time of meeting, agenda item)*

**Support network**

7. Could you tell me about the support you can access when doing patient and public engagement?

*E.g. team members, research community or institutional*

*Any local guidance, policies or legislation to influence your engagement strategies?*

8. What could be put in place to support your skills and increase confidence when engaging the public?

*How does patient and public engagement, as direct working with people, differ from your usual research role?*

9. How does patient and public engagement impact your workload?

**Impact**

10. What do/did you expect the impact of the engagement would be?

E.g. in a research project, you, as a researcher, or members of the public.

11. What contributions did you receive?

*In your opinion, was this worth it to you when compared with the time and resources dedicated to patient and public engagement?*

**Conclusion**

12. Reflecting on your engagement process, did anything happen that was a surprise to you?
13. That’s all the questions I had for you. Is there anything else you would like to mention or discuss?

*Stop the recording*

**Wrap up**

14. If you would like a copy of the findings when these become available, please let us know, and we will forward them to you.

*Thank the participant for their time.*
