# Supplementary material for: Bridging Lived and Expert Experience: A Qualitative Study Exploring Lived‐Experience Experts' and Researchers' Experiences of Public Engagement in Cancer Research
Source: Health Expect. 2026 Aug 31;29(5):e70848. doi: 10.1111/hex.70848 (PMC13527529; doi:10.1111/hex.70848)
Supplement: Supplementary file 2 [file HEX-29-e70848-s002.docx]

**Semi-structured topic guide for the lived-experience participant interview**

*The main questions are numbered with optional prompts in italics.*

**Warm-up discussion**

1. Could you tell me a bit about why you got interested in joining the community advisory board?

*Could you tell me how your engagement started? How did you find out about this opportunity? Any previous engagement or advocacy role?*

*How long did you serve? Is this still ongoing?*

**Engagement experience**

2. Could you tell me what your engagement looks like?

*How was your CAB environment?*

*E.g. one-off or multiple meetings, meeting length, at the stage of study (design, data collection, etc), incentives, setup (e.g. in-person, online, group discussion, survey).*

*Could you tell me about your induction? E.g. materials, training you received as a part of the role*

*Was the information provided in the language you could understand (lay language, medical terms etc)?*

*How does the CAB make decisions (e.g. is it consensus or majority rule)? How did they handle conflict and disagreements?*

*If engagement finished, was there any point when you were disinterested?*

3. Could you tell me more about how meetings were facilitated?

*How was your experience of leadership and facilitation during meetings?*

*How was your experience with researchers, e.g. did they provide information when they promised to?*

4. What worked well for you?

What did not work well for you? What could be done about it?

Was your engagement plan reasonable and delivered on time?

5. Could you name one of the best moments you had when being a part of the community advisory board? Could you now think about the worst?

**Public voice**

6. Could you tell me a bit more about your background? How did this background influence your engagement experience? (e.g. financial, education background, race, language skills, personal beliefs)?

*How was it taken into consideration by the research team? E.g. accessibility, arranging time and space based on your preferences, etc.*

7. Did you feel your community was represented?

**Connections with others**

8. Could you tell me about the networking with other members of the community advisory board or researchers?

*E.g. developing connections with others, new friendships, meetings outside scheduled sessions*

**Impact**

9. Did you feel you made a difference?

*Did you feel useful/ helpful to your community after being in CAB?*

*Did you feel your experience was valid and listened to?*

10. How to make you feel valued for your contribution?

**Conclusion**

11. Reflecting on your engagement process, did anything happen that was a surprise to you or that you did not expect?
12. That’s all the questions I had for you. Is there anything else you would like to mention or discuss?

*Stop the recording*

**Wrap up**

13. If you would like a copy of the findings when these become available, please let us know, and we will forward them to you.

*Thank the participant for their time.*
